# The Effect of Platinum Electrocatalyst on Membrane Degradation in Polymer Electrolyte Fuel Cells

**DOI:** 10.3390/membranes5040888

**Published:** 2015-12-08

**Authors:** Merit Bodner, Bernd Cermenek, Mija Rami, Viktor Hacker

**Affiliations:** Institute of Chemical Engineering and Environmental Technology, NAWI Graz, Graz University of Technology, Inffeldgasse 25C, Graz 8010, Austria; E-Mails: mija.rami@tugraz.at (M.R.); viktor.hacker@tugraz.at (V.H.)

**Keywords:** polymer electrolyte fuel cell, membrane degradation, fluoride emission rate, segmented cell, OCV conditions, relative humidity cycling, membrane resistance, proton conductivity

## Abstract

Membrane degradation is a severe factor limiting the lifetime of polymer electrolyte fuel cells. Therefore, obtaining a deeper knowledge is fundamental in order to establish fuel cells as competitive product. A segmented single cell was operated under open circuit voltage with alternating relative humidity. The influence of the catalyst layer on membrane degradation was evaluated by measuring a membrane without electrodes and a membrane-electrode-assembly under identical conditions. After 100 h of accelerated stress testing the proton conductivity of membrane samples near the anode and cathode was investigated by means of *ex situ* electrochemical impedance spectroscopy. The membrane sample near the cathode inlet exhibited twofold lower membrane resistance and a resulting twofold higher proton conductivity than the membrane sample near the anode inlet. The results from the fluoride ion analysis have shown that the presence of platinum reduces the fluoride emission rate; which supports conclusions drawn from the literature.

## 1. Introduction

Polymer electrolyte fuel cells are a promising approach to realise a sustainable power supply due to their high efficiency and flexibility. However, the lifetime and thus the commercialisation of fuel cells are still limited by degradation effects of both the electrodes and the membrane.

The mechanism of Nafion^®^ degradation has been well described previously [1,2,3]. Membrane degradation is accelerated under open circuit voltage (OCV) conditions. This is due to the correlation between the fluoride emission rate and hydrogen crossover [4]. Oxygen crossover has a similar effect, but the permeability of oxygen is lower than that of hydrogen [5]. The ionomer decomposes with the degradation front starting on the cathode side. Thereby, hydrogen crossover leads towards mixed potentials and thus a reduced open circuit voltage. Once the cathode ionomer has been consumed, both the hydrogen crossover and the fluoride emission rate reach plateaus until the anode ionomer starts to decompose [5].

Hydrogen peroxide formation has long been considered to be a major contributor to membrane degradation. Other than carbon corrosion, which is provoked by high potentials [6], it has been reported that the formation of hydrogen peroxide is favoured at anodic potential. Perfluorinated sulfonic acid (PFSA) membranes are fairly stable even in concentrated H_2_O_2_ solutions; yet degrade in the presence of metallic contaminations such as iron, aluminium, copper and titanium due to the formation of harmful radical in the presence of these metals [7,8]. Nafion electrolyte has been reported to only decompose in the presence of platinum electrocatalyst, hydrogen and oxygen in a setup with similar conditions as in a fuel cell [9]. On the other hand, Pt has also been reported to reduce the negative effect of hydrogen peroxide formation by decomposing it without the formation of harmful radicals [10]. Thus, *ex situ* experiments [9,10] and experiments with platinum implemented in the membrane [11] have shown that platinum electrocatalyst both enables and counteracts membrane degradation. In this work, the effect of the platinum electrocatalyst on the fuel cell operation was determined in a single cell setup.

## 2. Results

### 2.1. Accelerated Stress Test

**Figure 1:**
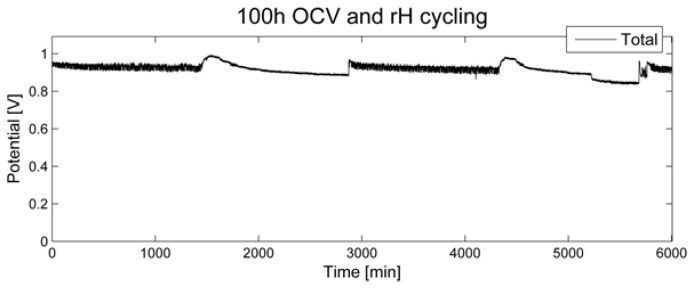
Open circuit voltage for 100 h of relative humidity cycling.

During 100 h of open circuit voltage and relative humidity (rH) cycling as accelerated stress test (AST), the voltage was recorded (Figure 1). No significant decrease over a time period of 100 h was noted. When switching to a relative humidity of 0%, a short period of increased OCV was recorded, followed by a slow decline. When switching back to a relative humidity of 80%, a similar but less pronounced effect was noted.

### 2.2. Electrochemical Characteristics of the Membrane-Electrode-Assembly

**Figure 2:**
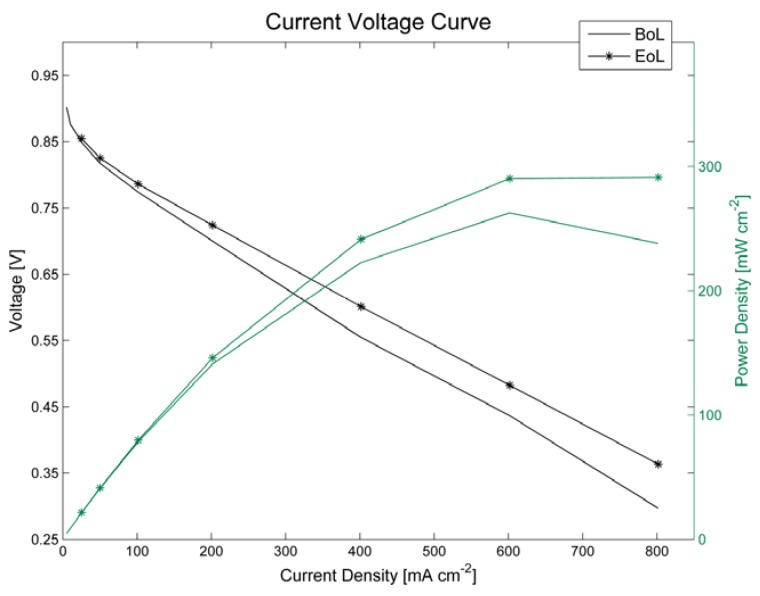
Polarisation curve of a single cell before (BoL) and after (EoL) 100 h at OCV and under humidity cycling.

Electrochemical characterisation at the beginning of lifetime (BoL) and at the end of lifetime (EoL) shows an improvement of the fuel cell properties in most aspects (Table 1). The membrane-electrode-assembly (MEA) shows an increase of power density (Figure 2), reduced hydrogen crossover and increased active cathode catalyst surface area. After 100 h of OCV conditions and relative humidity cycling, the membrane resistance is only slightly increased by approximately 0.54%. This is an indication for minor membrane degradation.

**Table 1:** Electrochemical characteristics of a single cell before (BoL) and after (EoL) 100 h at OCV and under humidity cycling.

Electrochemical Parameter	Before (BoL)	After (EoL)
Power density (mW cm ^−2^)	262.66	291.28
Membrane resistance (mΩ)	9.28	9.32
Hydrogen crossover current density (mA cm^−2^)	0.510	0.451
Active surface area (m^2^ g^−1^)	31.031	41.852

**Figure 3:**
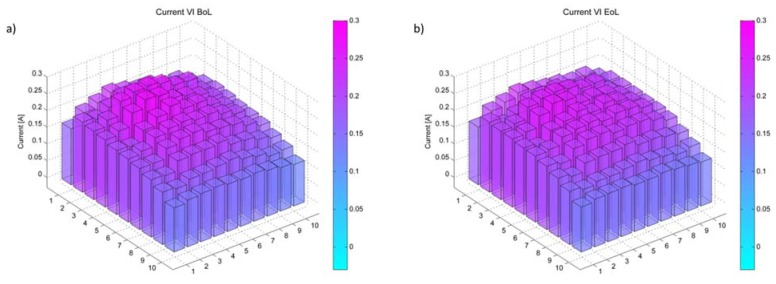
Local current at a total current of 20 A spatially resolved for a single cell (**a**) before (BoL) and (**b**) after (EoL) 100 h at OCV and under humidity cycling.

Most notably is, however, the strong increase of active catalyst surface area on the cathode side. The spatially resolved current shows a rather homogeneous distribution (Figure 3). Thus, this effect appears not to depend on local conditions near either electrode. This might be due to hydrogen, crossing over from the anode to the cathode. The presence of hydrogen on the cathode results in a chemical reduction of the cathode catalyst, thus increasing the active catalyst surface area. The uniform increase might be due to the thick membrane used in these experiments, possibly leading to a more homogeneous crossover. This can be explained by the effect of crossover on membrane degradation. A thinner membrane allows more hydrogen crossover which then locally accelerates membrane degradation in areas of higher hydrogen partial pressure, inducing a gradient in membrane thickness and thus a gradient in crossover.

In general, electrochemical characterisation has shown an improvement of the fuel cell parameters after 100 h of humidity cycling under OCV conditions.

### 2.3. Effect of the Electrodes on the Polymer Electrolyte Degradation

#### 2.3.1. Change of Membrane Resistance

**Figure 4:**
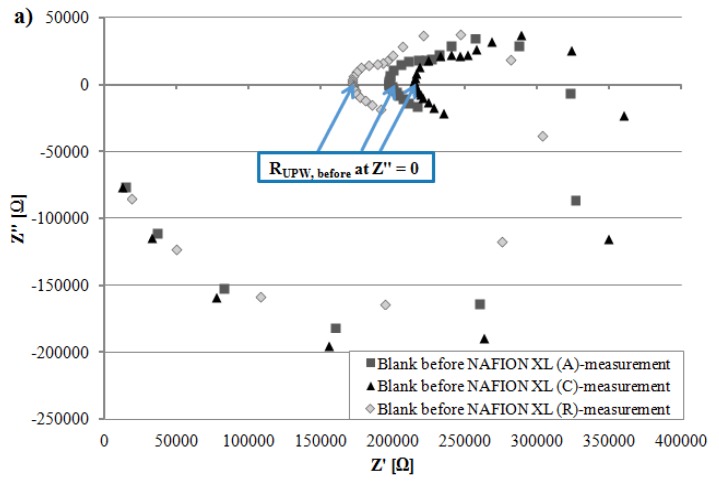

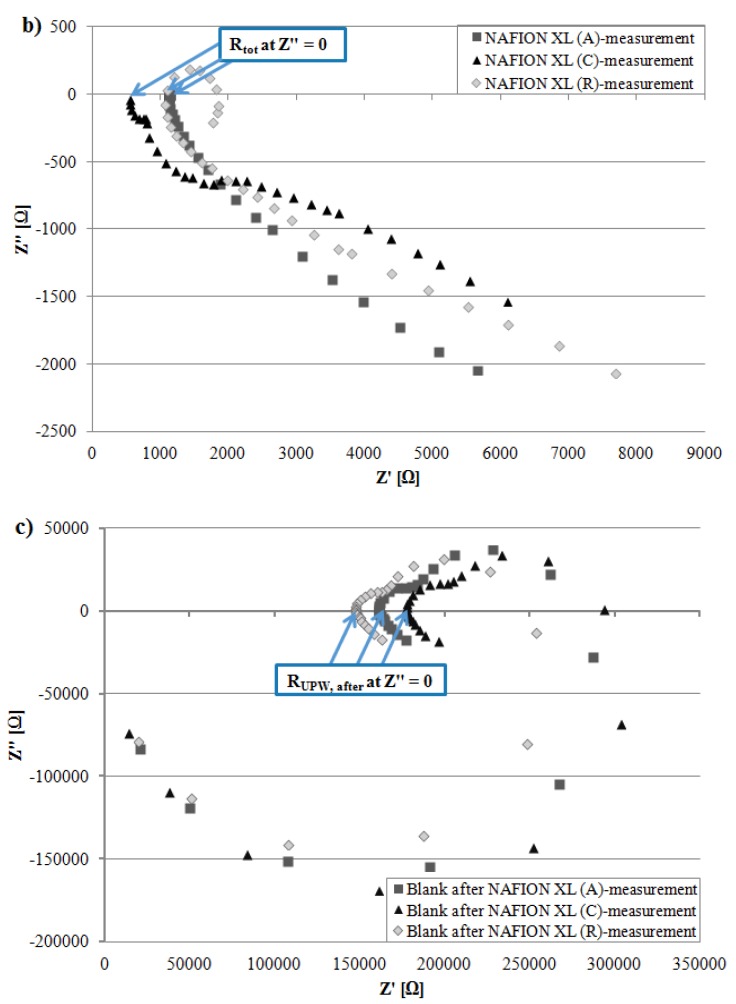
Comparison of (**a**) resistance from ultra-pure water (~18 MΩ·cm) R_UPW, before_ (**b**) measured resistance R_tot_ from NAFION^®^ XL (A), (C) and (R) (**c**) resistance from ultra-pure water R_UPW, after_.

Figure 4b depicts after 100 h of AST that the measured resistance R_tot_ of NAFION^®^ XL (A) near the anode inlet is only slightly higher and the measured resistance R_tot_ of NAFION^®^ XL (C) near the cathode inlet is lower than measured resistance R_tot_ of NAFION^®^ XL (R) used as reference (Table 2). The measured resistance R_tot_ denotes the sum of the inverse conductivity from respective NAFION^®^ XL membrane (σ_membrane_ = 1/R_membrane_) and ultra-pure water (σ_UPW_ = 1/R_UPW_), respectively.

**Table 2:** Determined measured parameters of NAFION^®^ XL (A = anode inlet), (C = cathode inlet) and (R = reference); ultra-pure water (UPW), width of membrane (W), thickness of membrane (T), distance between the inner sense electrodes (d), resistivity of membrane (ρ), in-plane proton conductivity of membrane (σ).

Sample	R_tot_ (Ω)	R_UPW, before_ (Ω)	R_UPW, after_ (Ω)	R_mem._ (Ω)	W (cm)	T (cm)	d (cm)	ρ (Ω∙cm)	σ (mS·cm^−1^)
NAFION XL (A)	1150	197837	161176	1158	1.0	0.0131	0.425	36	28.06
NAFION XL (R)	1098	172042	147256	1106	1.0	0.0131	0.425	34	29.33
NAFION XL (C)	557	214935	177533	558	1.0	0.0133	0.425	17	57.23

**Figure 5:**
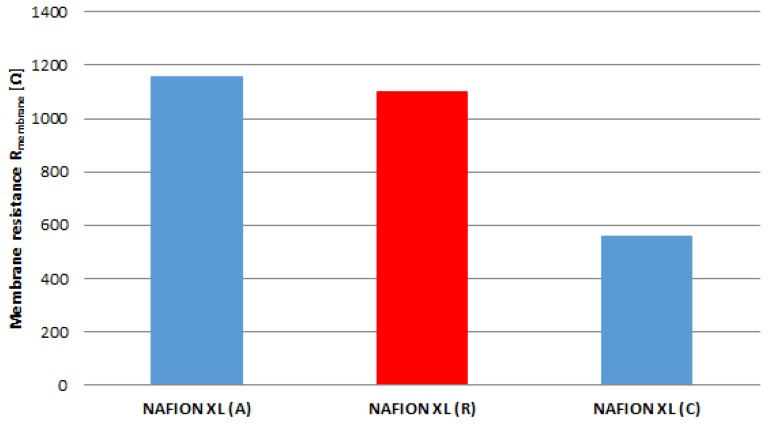
Membrane resistance (*ex*-*situ*) for the reference (R) and for samples near the anode inlet (A) and cathode inlet (C) after 100 h of AST.

The real membrane resistance R_membrane_ of samples (A), (C) and (R) corrected with the resistance of ultra-pure water after every EIS measurement (Equation (2)) is matched in Figure 5. It is evident that the real membrane resistance R_membrane_ of membrane sample (A) increases by approximately 5% and of membrane sample (C) decreases by approximately 49% compared to reference sample (R).

**Figure 6:**
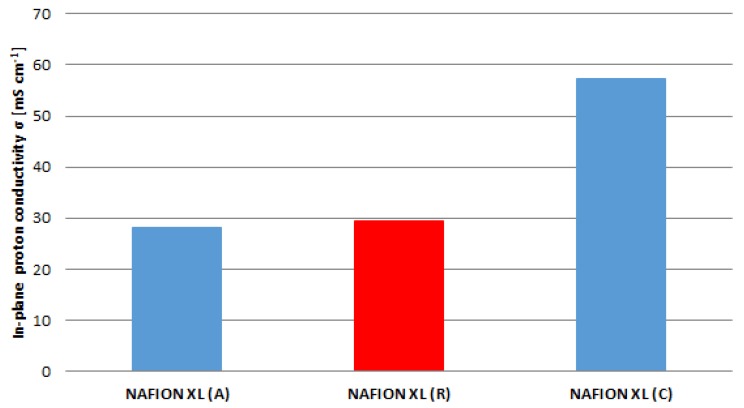
Proton conductivity (*ex*-*situ*) for the reference (R) and for samples near the anode inlet (A) and cathode inlet (C) after 100 h of AST.

The obtained results from Figure 5 are related to the results of Figure 6. The in-plane proton conductivity of membrane samples (A) and (C) exhibits a decrease of approximately 5% as well as an increase of approximately 49% in comparison with reference sample (R).

The membrane sample near the anode inlet (A) indicates a higher degradation rate than the membrane sample near the cathode inlet (C) after 100 h of AST. The determined measured parameters of all membrane samples are once more summarised in Table 2.

#### 2.3.2. Fluoride Emission Rate

**Figure 7:**
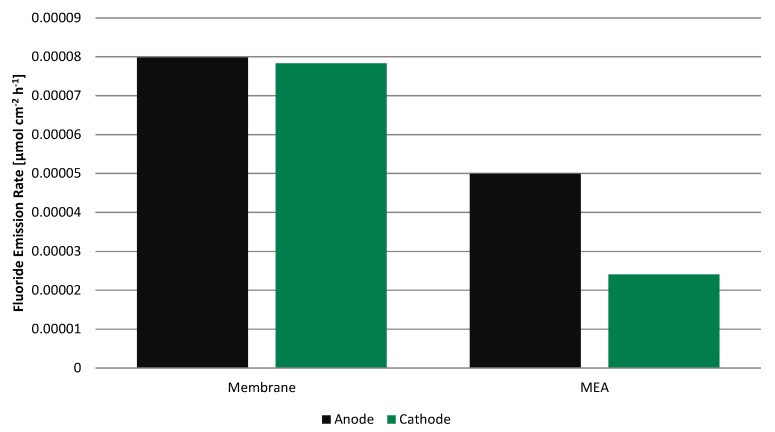
Fluoride emission rate during 100 h operation on anode and cathode side for the membrane alone and for the MEA.

Even though the fluoride concentration is low in all four samples, the fluoride emission rate (FER) (Figure 7) is clearly higher for the membrane without electrodes than for the MEA by a factor of 1.6 and 3.2 on the anode and cathode, respectively. Before adding total ionic strength adjustment buffer II (TISAB II), the pH was in the range of 5.45 to 5.68 for all samples and apparently independent of the fluoride concentration, always slightly higher on the cathode side.

#### 2.3.3. Off-Gas Analysis

**Figure 8:**
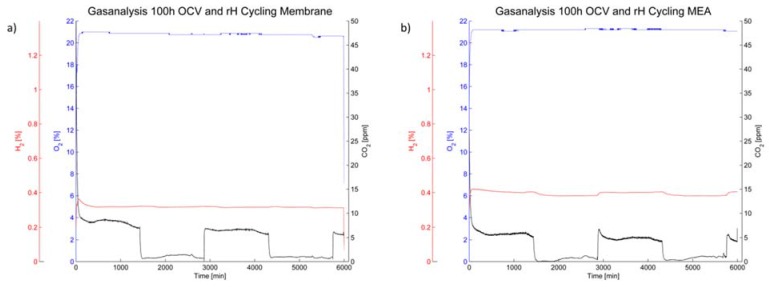
Gas analysis of the cathode off-gas for (**a**) the membrane without electrodes and (**b**) the membrane-electrode-assembly.

The off-gas analysis (Figure 8) shows a rather steady oxygen partial pressure during the 100 h of OCV operation. The hydrogen content is also steady during humidity cycling for the membrane (Figure 8a); however it increased during operation of the membrane-electrode-assembly and furthermore changes with humidity cycling (Figure 8b).

The CO_2_ content is, while rather low in general, dependent on the relative humidity in the cell. A higher carbon emission rate was noted under humid conditions for both the membrane without electrodes and the MEA. However, the level of CO_2_ in the cathode off-gas was higher for the membrane without electrodes than for the MEA (Figure 8).

## 3. Discussion

In Table 3, results at various conditions and with different materials are shown. Compared to the literature [1,4,5,7,12,13], the fluoride emission rate is low in all cases. More fluoride is released from the membrane without electrodes. This is rather unexpected compared to other findings [4]. However, a deceleration of membrane decomposition in the presence of platinum has been reported previously [10,11]. There, it is assumed that Pt catalyses the decomposition of H_2_O_2_ formed in case of gas crossover without the formation of harmful radicals.

**Table 3:** Comparision of the fluoride emission rate at different conditions.

Current	Temperature	Relative Humidity (%)	Total Duration	Membrane	Conditions	FER	Reference
0 (A)	65 (°C)	0–80 (%), 24 (h) interval	100 (h)	Nafion^®^ XL	H_2_/synth. Air	1.58·10^−4^ (µmol·cm^−2^·h^−1^)	this work
0 (A)	65 (°C)	0–80 (%), 24 (h) interval	100 (h)	Nafion^®^ XL MEA	H_2_/synth. Air	7.40·10^−5^ (µmol·cm^−2^·h^−1^)	this work
0 (A)	95 (°C)	50 (%)	200 (h)	PFSA-type MEA	H_2_/synth. Air	1·10^−5.5^–1·10^−4.5^ (gF·cm^−2^·h^−1^)*	[1]
0 (A)	90 (°C)	30 (%)	NA	Nafion 117	H_2_/O_2_	0.05 (µmol ·cm^−2^·h^−1^)	[4]
0 (A)	90 (°C)	30 (%)	NA	Nafion 117, anode only	H_2_/O_2_	2.5 (µmol·cm^−2^·h^−1^)	[4]
0 (A)	90 (°C)	30 (%)	NA	Nafion 117, cathode only	H_2_/O_2_	2.5 (µmol·cm^−2^·h^−1^)	[4]
0 (A)	90 (°C)	75 (%)	900 (h)	Gore PRIMEA^®^ 5510 CCM	H_2_/synth. Air	0–7.00 (µmol·cm^−2^·h^−1^)*	[5]
300 (mA·cm^−2^)	75 (°C)	0–100 (%), 10 (min) interval	441 (h)	Ion Power^®^ NR 212 MEA	H_2_/synth. Air	0.01–0.225 (µmol·cm^−2^·h^−1^) *	[12]
10 (mA·cm^−2^)	70 (°C)	0–100 (%), 10/40 (min) interval	625 (h) *	Gore™ 57 CCM	H_2_/synth. Air	0.1–6.9 (µmol·h^−1^) *	[13]

Note: * Taken from plot.

The performance of the fuel cell is ascending, despite of a minor increase of the membrane resistance. The improved electrochemical properties of the MEA after 100 h of AST might be due to the chemical reduction of Pt-oxides in the cathode catalyst by hydrogen, crossing over from the anode. Due to the relatively thick membrane, the crossover is homogeneous, since thinner membranes exhibit a higher hydrogen crossover, leading to a gradient in membrane degradation and thus a gradient in crossover.

After switching to dry operating conditions, the partial pressures of the reactant gases increase and the open circuit voltage surges. During relative humidity cycling, a slightly increased level of hydrogen was noted in the cathode off gas for the MEA. The level also increases at humid conditions for the MEA, whilst it remains unchanged for the membrane without electrodes.

The permeability of membranes towards gases is a function of temperature and humidity [14]. This might cause the fluctuating hydrogen crossover in the MEA experiment, but should be identical for both setups. Thus, the more likely reason is that this effect is somehow caused by the manufacturing process. Since both membrane and MEA were used as received, the latter cannot be precluded. Other than the membrane, the MEA had to undergo hot-pressing. This could have led to thermal degradation of the polymer electrolyte or a morphological change [3].

Additionally, the CO_2_ content in the cathode off-gas is, while rather low in comparison with the literature [15], clearly dependent on the relative gas humidity. This is expected to be caused by carbon corrosion of the carbon backing layer and catalyst support at high relative humidity. However, the carbon content is even higher in case of the membrane without electrodes and thus without carbon support materials. In this case, carbon may only be derived from two sources—the graphitic flow plates or the carbon from the proton conductive polymer. However, the carbon oxidation of graphitic bipolar plates is potential dependent and thus provoked by the positive potential at the air electrode [6]. Graphite oxidation is therefore only expected when using a membrane-electrode-assembly, since no voltage was recorded without the presence of platinum electrocatalyst. Thus, membrane degradation is left as source of the higher amount of carbon dioxide in the cathode off-gas.

The ex-situ EIS measurements show that NAFION^®^ XL sample (C) near the cathode inlet after 100 h of AST exhibits lower membrane resistance as well as higher in-plane proton conductivity than a NAFION^®^ XL sample (A) near the anode inlet after 100 h of AST and a reference sample (R) without stress, respectively. The better performance of NAFION^®^ XL sample (C) compared to the other membrane samples is probably due to the higher water content. As the cathode gas flow is higher, more water is transported into the cathode department and might accumulate near the inlet, leading to a gradient in proton conductivity. This assertion can be explained by means of the random network model as well as the cluster network model [16].

## 4. Conclusions

From the fluoride emission rate and the carbon dioxide release, it can be concluded that the presence of platinum electrocatalyst in the fuel cell reduces membrane degradation significantly. This is despite the higher hydrogen crossover in the MEA, which is apparently caused by the manufacturing process. Gas crossover is known to accelerate hydrogen peroxide formation. In the presence of platinum electrocatalyst, however, H_2_O_2_ appears to be decomposed without forming harmful radicals in a fuel cell setup as well as it has been reported in an ex-situ setup.

Hydrogen passing through the membrane is also responsible for the increased performance density and the homogeneous current distribution after 100 h of AST testing.

## 5. Experimental Section

A Nafion^®^ XL supported membrane without electrodes was implemented in a 25 cm² single cell with single serpentine flow fields on both sides and a segmented cathode. A S++ device for spatially resolved measurement of the current in 100 segments and of the temperature in 25 segments was used.

A membrane-electrode-assembly from IRD fuel cells A/S was used with an identical membrane and a catalyst loading of 0.2 mg·cm^−2^ on the anode and 0.4 mg·cm^−2^ on the cathode, respectively. The MEA was implemented in the same single cell as the membrane, underwent electrochemical characterisation and was operated under identical conditions. Both membrane and MEA underwent 100 h of AST testing. Afterwards, the cell with electrodes was again electrochemically characterised.

Effluent water was collected on both sides and the off-gas was analysed on the cathode side in both setups. Electrochemical characterisation, effluent water and off-gas analysis were performed as described previously [15].

### 5.1. Accelerated Stress Test

**Figure 9:**
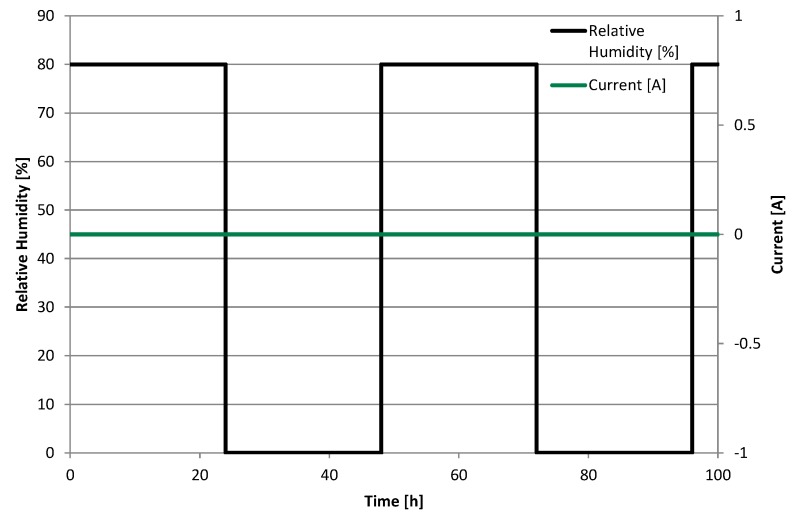
Accelerated Stress Test.

As an accelerated stress test (Figure 9), the cell was kept under OCV conditions at atmospheric pressure and was operated in pseudo-counter flow. One side, noted as anode, was fed with hydrogen (5.0) at a flow rate of 86.8 mL·min^−1^ and the other side, noted as cathode, was fed with synthetic air (5.0) at a flow rate of 275.3 mL·min^−1^. This represents a stoichiometry of 1.5 and 2.0 at a hypothetical current density of 0.3 A·cm^−2^ at anode and cathode, respectively. The temperature was held at 65 °C and the relative humidity was switched between 80% and 0% every 24 h for 100 h. Thereby, a combination of chemical and mechanical membrane degradation was induced. A hold at open circuit potential accelerates chemical membrane degradation [5], while relative humidity cycling causes swelling and shrinkage of the membrane, thus inducing mechanical stress [12]. Combining mechanical and chemical stressors have been shown to have overlapping effects, resulting in a severe reduction of durability for example for an MEA being operated under normal operation conditions accompanied by relative humidity cycling [17].

Gases were humidified using bubblers with monitoring of the relative humidity in the heated cathode feed line using a SHT75 temperature and humidity sensor from Sensirion AG. It was evident, that the relative humidity declined rapidly after switching to dry operation and increased rapidly after switching back to humidified operation. Here, “rapidly” means in a time range of approximately 10 min.

### 5.2. Determination of Fluoride Ion Using Fluoride Ion Selective Electrode

The Fluoride Ion Selective Electrode (F^−^-ISE) is considered to be the simplest and most reliable technique to determine the concentration of fluoride ions in a solution [18]. Measurements with a F^−^-ISE are rapid (the response time being of order of 10^−2^ s to 2 min), non-destructive, easy and requiring simple equipment [19]. In this work the concentration of fluoride ions in fuel cell effluent water was measured by means of direct potentiometry using a F^−^-ISE.

#### 5.2.1. Apparatus

An Orion 5-Star pH/ISE/DO/conductivity meter was used to measure the potential of the Thermo Scientific Orion^®^ 9609BNWP Fluoride Combination Ion Selective Electrode at room temperature.

#### 5.2.2. Reagents

Chemicals of analytical grade were used without further purification. Series of standard solutions (conc. F^−^ ion; 0.02, 0.2 ppm) were prepared by suitable dilution of 2 ppm F^−^ with Total Ionic Strength Adjustment Buffer II standard. Samples have been prepared using TISAB II solution, which provides a constant background ionic strength, decomplexes fluoride ions and adjusts the solution pH between 5.0 and 5.5 [20]. All solutions were prepared from water which had been both deionized and distilled. Thermo Scientific Orion^®^ 9609BNWP Fluoride Combination Ion Selective Electrode was filled with Optimum Results A™ ISE filling solution from Thermo Scientific Orion.

#### 5.2.3. Measurement Procedure

Fluoride Combination Ion Selective Electrode was calibrated using three standard solutions; conc. F^−^ ion 0.02, 0.2 and 2.0 ppm. After obtaining an electrode slope value between −54 and −60 mV the concentration of fluoride ions in given samples has been measured. Samples were prepared by mixing the fuel cell effluent water and TISAB II in a ratio of one to one. The concentration of fluoride ions in the sample was read directly from the device display. After every measurement the electrode was rinsed thoroughly with water and calibrated anew. In the time between measurements the electrode was stored in the standard solution with the lowest concentration of fluoride ion.

### 5.3. Determination of in-Plane Proton Conductivity of NAFION^®^ XL Membrane Using Electrochemical Impedance Spectroscopy

The membrane resistance of three pieces of NAFION^®^ XL (A, C, R) was determined by means of electrochemical impedance spectroscopy (EIS) using a Conductivity Clamp (Bekktech BT110 LLC, Scribner Associates, Southern Pines, NC, USA) to calculate the in-plane proton conductivity of each membrane. The denotations A, C and R in bracket mean pieces of membrane from near the anode inlet, cathode inlet and a piece of an untreated membrane used as reference.

For the test procedure, two pieces of the NAFION^®^ XL membrane after AST were cut out at sizes of 2.5 × 1.0 cm with a scalpel from the anode inlet and cathode inlet, respectively. Further, the respective membrane sample was incorporated in the BT110 Conductivity Clamp which was connected with a GAMRY Instruments INTERFACE 1000 Potentiostat/Galvanostat/ZRA in a four-electrode arrangement. The BT-110 Conductivity Clamp including membrane was hung in a heated surface-grinding cell filled with ultra-pure water (~18 MΩ·cm) and parameters were adjusted in GAMRY software program for EIS measurements.

Generally, following measuring process was realised for every membrane sample:

- before: 5 EIS measurements of ultra-pure water (without membrane)
- 5 EIS measurements of respective NAFION^®^ XL membrane
- after: 5 EIS measurements of ultra-pure water (without membrane)

All EIS measurements were carried out at 65 °C in potentiostatic mode and 5 points per decade were recorded. Before the EIS measurements were started, the open circuit potential (OCP) was measured for 50 s. A sinusoidal alternating current (AC) voltage of 50 mV (rms) was applied on the electrochemical system (BT110 Conductivity Clamp including membrane) in a frequency range of 0.1–100,000 Hz and the sinusoidal AC current was measured resulting the impedance Z of the membrane. From the resulting Nyquist-diagram, the frequency-independent measured resistance R_tot_ of respective membrane was read in high frequency range (HFR) at the measurement point which was sliced with the *x*-axis corresponding to the real part of impedance Z′ at imaginary part of impedance Z″ equals zero (*y*-axis) (Figure 4). The value of the measured resistance R_tot_ was used to assign the real membrane resistance R_membrane_ of respective NAFION^®^ XL. The charge transport (H^+^) occurs not only through the membrane, but also via the electrolyte (~18 MΩ·cm water). Therefore, the electrolyte resistance R_UPW, after_ should be considered for the calculation of proton conductivity of respective membrane (Equation (1)) [21,22].

(1) $$\frac{1}{R_{tot}}=\;\frac{1}{R_{membrane}}+\;\frac{1}{R_{UPW,after}}$$

The membrane resistance *R_membrane_* is specified in Equation (2) [21,22].

(2) $$R_{membrane}=\;\frac{1}{\frac{1}{R_{tot}}-\;\frac{1}{R_{UPW,after}}}$$

For the determination of in-plane proton conductivity, the thickness T of the respective NAFION^®^ XL membrane in wet state was measured by a micrometer screw (10-fold determination) after measurement process. Equation (3) shows how the in-plane proton conductivity of respective membrane was calculated [21,22].

(3) $$\sigma_{membrane}=\;\frac{d}{R_{membrane}\cdot T\cdot W}$$

The meaning of parameters from Equation (3) was already described in Table 2 (Section 2.3.1).

## Abbreviations

AST: accelerated stress test
BoL: beginning of lifetime
EIS: electrochemical impedance spectroscopy
EoL: end of lifetime
FER: fluoride emission rate
F−-ISE: fluoride ion selective electrode
MEA: membrane-electrode-assembly
OCP: open circuit potential
OCV: open circuit voltage
PFSA: perfluorinated sulfonic acid
rH: relative humidity
TISAB: total ionic strength adjustment buffer
UPW: ultra-pure water

## References

[1] Coms F.D. (2008). The chemistry of fuel cell membrane chemical degradation. ECS Trans..

[2] Collier A., Wang H., Ziyuan X., Zhang J., Wilkinson D. (2006). Degradation of polymer electrolyte membranes. Int. J. Hydrog. Energy.

[3] Samms S.R. (1996). Thermal stability of Nafion^®^ in simulated fuel cell environments. J. Electrochem. Soc..

[4] Mittal V.O., Russell-Kunz H., Fenton J.M. (2006). Is H_2_O_2_ Involved in the membrane degradation mechanism in PEMFC?. Electrochem. Solid-State Lett..

[5] Kundu S., Fowler M.W., Simon L.C., Abouatallah R., Beydokhti N. (2008). Degradation analysis and modeling of reinforced catalyst coated membranes operated under OCV conditions. J. Power Sources.

[6] Heinzel A., Mahlendorf F., Jansen C. (2009). Fuel cells—Proton-exchange membrane fuel cells bipolar plates. Encycl. Electrochem. Power Sources.

[7] Aarhaug T.A., Svensson A.M. (2006). Degradation rates of PEM fuel cells running at open circuit voltage. ECS Trans..

[8] Li H., Tsay K., Wang H., Shen J., Wu S., Zhang J., Jiab N., Wesselb S., Abouatallahc R., Joosc N. (2010). Durability of PEM fuel cell cathode in the presence of Fe^3+^ and Al^3+^. J. Power Sources.

[9] Aoki M., Uchida H., Watanabe M. (2005). Novel evaluation method for degradation rate of polymer electrolytes in fuel cells. Electrochem. Commun..

[10] Aoki M., Uchida H., Watanabe M. (2006). Decomposition mechanism of perfluorosulfonic acid electrolyte in polymer electrolyte fuel cells. Electrochem. Commun..

[11] Macauley N., Wong K.H., Watson M., Kjeang E. (2015). Favorable effect of in-situ generated platinum in the membrane on fuel cell membrane durability. J. Power Sources.

[12] Vengatesan S., Fowler M.W., Yuan X.-Z., Wang H. (2011). Diagnosis of MEA degradation under accelerated relative humidity cycling. J. Power Sources.

[13] Panha K., Fowler M., Yuan X.-Z., Wang H. (2012). Accelerated durability testing via reactants relative humidity cycling on PEM fuel cells. Appl. Energy.

[14] Catalano J., Myezwa T., de Angelis M.G., Baschetti M.G., Sarti G.C. (2012). The effect of relative humidity on the gas permeability and swelling in PFSI membranes. Int. J. Hydrog. Energy.

[15] Bodner M., Hochenauer C., Hacker V. (2015). Effect of pinhole location on degradation in polymer electrolyte fuel cells. J. Power Sources.

[16] Smitha B., Sridhar S., Khan A.A. (2005). Solid polymer electrolyte membranes for fuel cell applications—A review. J. Membr. Sci..

[17] Gittleman C.S., Coms F.D., Lai Y. (2012). Membrane durability. Polym. Electrolyte Fuel Cell Degrad..

[18] Sunitha V., Reddy B.M. (2014). Determination of fluoride concentration in ground water by ion selective electrode. Int. J. Curr. Res. Aca Rev..

[19] Alcacer L., Barbosa M.R., Almeida R.A., Marzagao M.F. (1973). On the preparation of ion-selective electrodes. Rev. Port. Quím..

[20] Pakalns P. (1983). Non-ionic detergent as a complexing agent in the determination of fluoride with the fluoride electrode. Microchim. Acta.

[21] Dedmond E., Cooper K. Application Note—Effect of Solution Conductivity on in-Plane Membrane Conductivity Measurement. http://www.scribner.com/files/knowledgebase.

[22] Ranacher C., Resel R., Moni P., Cermenek B., Hacker V., Coclite A.M. (2015). Layered nanostructures in proton conductive polymers obtained by initiated chemical vapor deposition. Macromolecules.

